# Clustering of HIV-1 Subtypes Based on gp120 V3 Loop electrostatic properties

**DOI:** 10.1186/2046-1682-5-3

**Published:** 2012-02-07

**Authors:** Aliana López de Victoria, Chris A Kieslich, Apostolos K Rizos, Elias Krambovitis, Dimitrios Morikis

**Affiliations:** 1 Department of Bioengineering, University of California, Riverside 92521, USA; 2 Department of Chemistry, University of Crete and Foundation for Research and Technology-Hellas, FORTH-IESL, GR-71003, Heraklion, Crete, Greece; 3 Department of Veterinary Medicine, University of Thessaly, Karditsa, Greece

**Keywords:** HIV-1, protein-receptor interactions, Poisson-Boltzmann electrostatics, electrostatic similarity distance, electrostatic clustering

## Abstract

**Background:**

The V3 loop of the glycoprotein gp120 of HIV-1 plays an important role in viral entry into cells by utilizing as coreceptor CCR5 or CXCR4, and is implicated in the phenotypic tropisms of HIV viruses. It has been hypothesized that the interaction between the V3 loop and CCR5 or CXCR4 is mediated by electrostatics. We have performed hierarchical clustering analysis of the spatial distributions of electrostatic potentials and charges of V3 loop structures containing consensus sequences of HIV-1 subtypes.

**Results:**

Although the majority of consensus sequences have a net charge of +3, the spatial distribution of their electrostatic potentials and charges may be a discriminating factor for binding and infectivity. This is demonstrated by the formation of several small subclusters, within major clusters, which indicates common origin but distinct spatial details of electrostatic properties. Some of this information may be present, in a coarse manner, in clustering of sequences, but the spatial details are largely lost. We show the effect of ionic strength on clustering of electrostatic potentials, information that is not present in clustering of charges or sequences. We also make correlations between clustering of electrostatic potentials and net charge, coreceptor selectivity, global prevalence, and geographic distribution. Finally, we interpret coreceptor selectivity based on the N^6^X^7^T^8^|S^8^X^9 ^sequence glycosylation motif, the specific positive charge location according to the 11/24/25 rule, and the overall charge and electrostatic potential distribution.

**Conclusions:**

We propose that in addition to the sequence and the net charge of the V3 loop of each subtype, the spatial distributions of electrostatic potentials and charges may also be important factors for receptor recognition and binding and subsequent viral entry into cells. This implies that the overall electrostatic potential is responsible for long-range recognition of the V3 loop with coreceptors CCR5/CXCR4, whereas the charge distribution contributes to the specific short-range interactions responsible for the formation of the bound complex. We also propose a scheme for coreceptor selectivity based on the sequence glycosylation motif, the 11/24/25 rule, and net charge.

## Background

HIV-1 entry into the host cell is mediated by the viral envelope glycoprotein gp120 associated with gp41 and involves on the host cell surface the CD4 molecule together with the CCR5 or CXCR4 receptor [1,2]. Upon CD4 binding, a conformational change is induced in gp120, exposing a region that can interact with CCR5 or CXCR4 [2]. CCR5 and CXCR4 belong to the chemokine receptor family, which is part of the G-protein couple receptor (GPCR) superfamily, a large group of membrane proteins characterized by seven transmembrane α-helices and four extracellular and four intracellular domains. CD4 binding also can induce further conformational changes in the envelope glycoprotein, exposing a glycine rich region of gp41 which is involved in membrane fusion [3,4].

The envelope glycoprotein gp120 is composed of 400-410 amino acids including 5 variable regions (V1-V5) [2,5,6]. The third variable region of gp120 forms a loop, called the V3 loop, and is composed of 31-39 amino acids. The V3 loop is closed by a disulfide bridge formed by two cysteines and is positively charged. It consists of three distinct regions: the base (closer to the core of the protein), the tip at the opposite end, and the stem between the base and the tip. The V3 loop is implicated in the phenotypic tropisms of HIV viruses, playing an important role in viral entry by utilizing as coreceptor CCR5 or CXCR4. Viruses utilizing CCR5 are referred to as R5 and are preferentially transmitted, whereas those utilizing CXCR4 are associated with disease progression and are referred to as X4. Considering that HIV viruses undergo mutations at very high rates, it is not unusual for several variants to exist in a given patient sample [7,8].

It has been suggested that when amino acids at positions 11 and/or 25 of the V3 loop are positively charged, the virus shows preference for selecting CXCR4 as coreceptor, and when the amino acid at position 11 is uncharged or negatively charged and at position 25 is negatively charged, the virus shows preference for CCR5 coreceptor [8-12]. This means that charge switch to positive at positions 11 or 25 suggests switch of coreceptor selection to CXCR4. It has also been suggested that, besides amino acids 11 and 25, amino acid 24 is also involved in coreceptor selection, with the proposition of the so-called "11/24/25" rule [12]. This rule states that positively charged amino acids at one or more of positions 11, 24 or 25 suggest an X4 virus.

**Figure 1:**
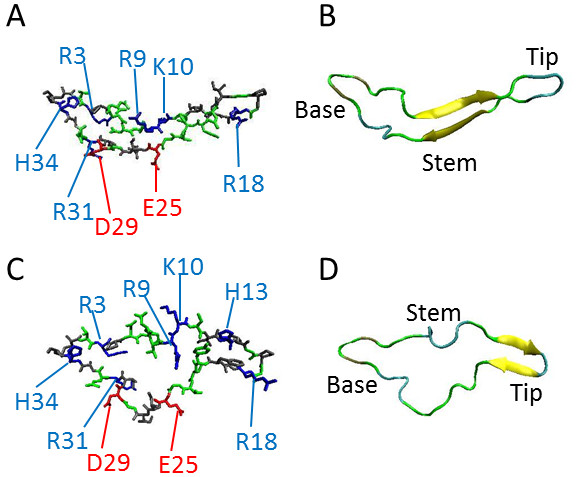
**Molecular models of the V3 loop**. (A) Stick representation of backbone and side chains using the gp120 structure with PDB Code 2QAD. (B) Ribbon representation of backbone using the structure with PDB Code 2QAD. (C) Stick representation of backbone and side chains using the gp120 structure with PDB Code 2B4C. (D) Ribbon representation of backbone using the gp120 structure with PDB Code 2B4C. The color code for (A) and (C) is: blue, positively charged; red, negatively charged; green, polar; gray, nonpolar. The color code for (B) and (D) is according to secondary structure. Images were generated using VMD [78].

The V3 loop is solvent exposed, highly charged, and highly dynamic. Its dynamic character is indicated by the fact that the V3 loop is absent in many crystallographic structures because of lack of resolved electron density. In two available crystallographic structures in which gp120 is stabilized because of multicomponent complex formation, the V3 loop is structurally resolved but with different secondary structure content ([3,6]; Figure 1). Several studies have demonstrated that the V3 loop interacts with the N-terminal extra-cellular domain of CCR5 (CCR5-Nt) and the extracellular loop 2 (ECL2) [6]. Post-translational modifications by the addition of sulfate groups in two or three of the tyrosines of CCR5-Nt have been shown to be essential in the interaction with gp120 [13-15]. The physicochemical mechanism of the gp120:CCR5 interaction is not well understood. Earlier studies have proposed that the interaction between CCR5-Nt and V3 loop is driven by electrostatics, between a highly positive V3 loop and a highly negative CCR5-Nt [16-19]. We have previously proposed a correlation between the strength of electrostatic potential with binding affinities and inhibitory activities for several V3 loop-derived peptides [18]. Another study of V3 loop chimeras has shown that their ability to bind CCR5 is affected by the amino acid composition and charge [11].

The diversity of HIV-1 presents a major challenge in the development of effective treatments. Currently, HIV-1 strains are divided into three distinct genetic groups: M (major), N (non-major, non-outlier), and O (outlier), with variants within group M being responsible for the majority of the infected population. This group is further divided based on the sequence variability of its *env *and *gag *genes [7] into ten subtypes or clades, named A through K, and circulation recombinant forms (CRFs). Differences in coreceptor usage, geographical distribution and global prevalence have been demonstrated for several of the identified subtypes [19-22].

In this study we have modeled the V3 loop of several HIV-1 subtypes using the available two crystal structures with intact V3 loop as templates [3,6] and consensus sequences, which were obtained from the HIV Databases of the Los Alamos National Laboratory [23]. We have performed computational studies to cluster the various subtypes according to similarities of the spatial distributions of their electrostatic potentials and the spatial distributions of their charges. The spatial distributions of individual charges are responsible for generating the spatial distributions of electrostatic potentials, while taking into account dielectric and ionic screening. We have analyzed the resulting clusters to determine correlations between the electrostatic potential distributions and charge distributions with net charge, epidemiological data such as global prevalence and geographical distribution, and coreceptor selection. We have also generated sequence alignment and sequence similarity clusters for all the V3 loop subtypes. Our goal was to perform a clustering analysis of the gp120 V3 loop of HIV-1 at various levels of refinement, based on sequence, net charge, and spatial distribution of electrostatic potential and charge. The electrostatic clustering analysis may be useful in much-needed vaccine, vaccine adjuvant, or inhibitor design against HIV-1 infection [24-26].

## Methods

Our computational framework AESOP (Analysis of Electrostatic Potentials Of Proteins) [27-31] was used to generate theoretical structures of several V3 loop subtypes, to calculate electrostatic potentials, and to cluster their respective spatial distributions of electrostatic potentials. We have also performed clustering analysis of V3 loop subtypes according to their charge distributions and sequence similarities.

### V3 loop structural templates

We used the coordinates of two Protein Data Bank (PDB [32]) files in which the V3 loop was intact, as structural templates. The PDB codes are 2B4C[5] and 2QAD [6], both from subtype B. In 2B4C, the gp120 core with V3 isolate JR-FL was complexed to CD4 (N terminal two-domain fragment) and the antigen-binding fragment (Fab) of the X5 antibody. In 2QAD, gp120 was in complex with CD4 and a functionally sulfated antibody, 412d. From both structures, we have retained only the coordinates of the V3 loop for our study. The V3 loop in both structures starts at position 296 and ends at position 331. In the case of 2B4C four amino acids have double conformations, from which conformation A was retained. In both structures amino acids 310-311 are missing while two amino acids occupy position 322. We have renumbered the atoms and amino acids starting from position 1 and ending in position 35, using Swiss-PDB Viewer (SPDBV, [33]).

### V3 loop subtype consensus sequences

HIV-1 sequences are deposited in the HIV Databases of the Los Alamos National Laboratory [[23]; http://www.hiv.lanl.gov]. Using tools within the database we extracted consensus sequences for the V3 loop of HIV-1. For our study, we isolated the amino acid sequences between and including the first and last cysteines of the V3 loop. The Sequence Search Interface Tool was first used to obtain nucleotide sequences for HIV-1 subtypes. Within this search tool, the parameters selected were: subtype (for example, subtype A), virus (HIV-1), and genomic region (V3). The search result file is the input file for the ElimDupes tool, which compares all the sequences and eliminates any duplicates. A cutoff of 93% DNA sequence identity of the *env *gene was used. The unique sequences file was used as the input file for the HIValign tool, which aligns the sequences based on curated alignments within the database using the Hidden Markov Model (HMM) method. Several options were selected for this tool: align the sequences by HMM, codon-align the sequences, and translate to amino acid. The Simple Consensus Maker tool was then used to obtain a consensus sequence, with the resulting file from HIValign being used as the input file. The default parameters were kept, resulting in an alignment sequence with the first sequence identified as the consensus.

This procedure was done for each subtype and groups N and O and the results of consensus sequence alignment are shown in Table 1. Subtype A includes sub-subtypes A1 and A2, subtype F includes sub-subtypes F1 and F2, and subtype CPX includes the 11 cpx subtypes available in the database. The consensus for subtype D resulted in 33 amino acid sequence, because of gaps at positions 24-25. To equalize the length of the D subtype with the 35-amino acid length of the rest of the subtypes, we calculated amino acid frequencies at positions 24-25 of the D subtype and chose the amino acids with the second highest frequency in the alignments (gaps being the highest frequency). These amino acids were lysine at position 24 and asparagine at position 25 (Table 1). Subtype J and group O contained two amino acids with the exact same frequency at a particular location. In the case of subtype J, the amino acids where isoleucine and leucine; and for group O, the amino acid was glutamic acid and a gap (introduced by the alignment). For subtype J, isoleucine was selected and for group O, glutamic acid was selected. Subtypes B and K have the same consensus sequences, and subtypes CPX and H also share identical consensus sequences. Subtypes AB, AE, AG, and CPX are circulating recombinant forms (CRFs).

**Table 1:** Alignment of the year 2009 consensus sequences of the V3 loop.

Subtype	Sequence	Charge
	**123456789012345678901234567890123456**	
2B4C	CTRPNQNTRKSIHIGPGRAFYTTG-EIIGDIRQAHC	3
2QAD	CTRPNNNTRKSINIGPGRALYTTG-EIIGDIRQAHC	3
A	CTRPNNNTRKSVRIGPGQAFYATG-DIIGDIRQAHC	3
AB	CIRPGNNTRTSIRIGPGQTFYATG-DVIGDIRQAHC	2
AE	CTRPSNNTRTSITIGPGQVFYRTG-DIIGDIRKAYC	3
AG	CTRPNNNTRKSVRIGPGQTFYATG-DIIGDIRQAHC	3
B	CTRPNNNTRKSIHIGPGRAFYATG-DIIGDIRQAHC	3
C	CTRPNNNTRKSIRIGPGQTFYATG-DIIGDIRQAHC	3
CPX	CTRPNNNTRKSIHIGPGQAFYATG-DIIGDIRQAHC	2
D	CTRPYNNTRQSTHIGPGQALYTT---IIGDIRQAHC	2
D35	CTRPYNNTRQSTHIGPGQALYTTK-NIIGDIRQAHC	3
F	CTRPNNNTRKSIHLGPGQAFYATG-DIIGDIRKAHC	3
G	CTRPNNNTRKSIRIGPGQAFYATG-DIIGDIRQAHC	3
H	CTRPNNNTRKSIHIGPGQAFYATG-DIIGDIRQAHC	2
J	CIRPANNTRKGIHIGPGQVLYATG-EIIGDIRQAHC	2
K	CTRPNNNTRKSIHIGPGRAFYATG-DIIGDIRQAHC	3
N	CTRPGNNTGGQVQIGPAMTFYNIE-KIVGDIRQAHC	1
O	CERPGNNTVQEIKIGP-MAWYSMGLEENNNSRAAYC	-1

V3 loop subtype consensus sequences were obtained using the tools available within the Los Alamos National Laboratory Database ([23]; http://www.hiv.lanl.gov), except for sequences 2B4C and 2QAD, which are from the crystal structures deposited at the PDB. Some subtype consensus sequences were identical: CPX with H, and B with K. Subtypes AB, AE, AG, and CPX are circulating recombinant forms (CRFs). The construction of D35 from D is described in Methods. Gaps are introduced by the alignment.

The program Modeller [9v6, 34] was used to create homology models of all subtypes, using the two crystal structures as templates, with the modifications described above. The default optimization and refinement protocol of Modeller was used to generate single models, optimized with conjugate gradients and molecular dynamics-based simulated annealing.

### Clustering of electrostatic potentials

The use of similarity measures for clustering of electrostatic (and other physicochemical) properties is a topic of chemistry and drug design research [35-38]. Clustering of electrostatic potentials of protein families has been introduced by Wade and coworkers [39-45], including software tools under the name PIPSA [39,40,43,44], and subsequently used or extended by others, including our group [27-31,46-51]. This type of analysis depicts electrostatic similarities of proteins, which can be correlated to biological properties and functions. For our analysis we used the AESOP computational framework [27-31], which provides a platform for elucidating the role of electrostatics, and more specifically the role of ionizable amino acids, in protein association. This is accomplished using theoretical alanine scan or other mutagenesis, in which electrostatic properties are perturbed by systematically removing ionizable amino acids [27-31,48,49,51]. The effects of these perturbations are then quantified through the use of electrostatic similarity clustering and electrostatic free energies of association, to give insights into the contributions of ionizable amino acids in both recognition and binding [27,28,30,31,48,49,51]. Since electrostatics is also known to be an important aspect of protein dynamics and evolution, AESOP also has utilities for analyzing the electrostatics of molecular dynamics trajectories [28] and homologous proteins/protein domains [31,47,50].

Poisson-Boltzmann electrostatic calculations and hierarchical clustering analysis were performed as described elsewhere [27-30]. The program PDB2PQR [52] was used to prepare the V3 loop coordinates for electrostatic calculations by including van der Waals radii and partial charges for all atoms according to the PARSE forcefield [53]. Electrostatic potentials were calculated using the Adaptive Poisson Boltzmann Solver (APBS [54]) and the linearized form of the Poisson-Boltzmann equation. A box with 129 × 129 × 129 grid points was used. The box dimensions were: 70 Å × 70 Å × 75 Å and 50 Å × 50 Å × 55 Å for 0 and 150 mM, respectively, for subtypes from the template 2B4C; and 60 Å × 70 Å × 75 Å and 50 Å × 50 Å × 50 Å for 0 and 150 mM, respectively, for subtypes from the template 2QAD. Different box sizes were used for 0 mM and 150 mM calculations to assure maximum resolution while including optimal number of grid points with electrostatic potential values within and about ± 1 k_B_T/e. The molecular surface was calculated using a probe sphere with a radius of 1.4 Å, representing a water molecule. The dielectric coefficients were set to 2 and 78.54 for the protein interior and solvent, respectively. The ion accessibility surface was calculated using a probe sphere with radius of 2.0 Å, representing monovalent counterions. Calculations were repeated with ionic strengths corresponding to 0 mM salt concentration (representing Coulombic interactions unscreened by counterions) and 150 mM (representing physiological ionic strength in serum). A total of 36 calculations were performed for the consensus sequence structures generated from each of the two templates and for the template (crystal) structures.

Electrostatic similarity distances (ESDs) were calculated according to

(1) $$ESD_{a,b}=\frac{1}{N}\underset{i,j,k}{\sum }\frac{\left|\Phi_{a}\left(i,j,k\right)-\Phi_{b}\left(i,j,k\right)\right|}{\max\left\{\left|\Phi_{a}\left(i,j,k\right)\right|,\left|\Phi_{b}\left(i,j,k\right)\right|\right\}}$$

where Φ_a _and Φ_b _are the electrostatic potentials of proteins a and b at grid point (i, j, k) and N is the total number of grid points. This error-type relation compares the spatial distributions of electrostatic potentials of pairs of proteins. A matrix of 18 × 18 ESDs was created corresponding to the HIV-1 subtype structures. The normalization factor of the denominator assures small values in the vicinity of the 0-2 range, with 0 corresponding to identical spatial distributions of electrostatic potentials and 2 to totally different. Four matrices were constructed for two sets of structures (from two templates), with electrostatic potentials calculated at two ionic strength values. Each matrix was analyzed separately. Visualization of the spatial distributions of electrostatic potentials, as isopotential contour surfaces, was accomplished using the program Chimera [55].

The ESD shown above was also applied to cluster subtype sequences based on charge distribution maps using APBS. Hierarchical clustering analysis was performed using the hclust function of R. The clustered data were plotted as dendrograms using the language and statistical computing environment R (Foundation for Statistical Computing: Vienna, Austria, 2009. http://www.R-project.org).

### Clustering of subtype sequences

Alignment for all HIV-1 subtype sequences of Table 1 was performed using ClustalW2 [56]. The score matrix generated by ClustalW2 was used as the input distance file to create a clustering dendrogram using the linkage function of MatLab (The MathWorks Inc., Natick, MA).

## Results and discussion

### Importance of V3 loop variability and charges for viral infection

HIV is characterized by its ability to frequently mutate as evidenced by the large number of different isolates and by sequence diversity. A variability "hotspot" is the V3 loop which is implicated in a number of important functions including coreceptor usage during cell entry. Despite its hypervariable nature, V3 retains a basic function, that to interact and to modulate its preferential usage of CCR5 and CXCR4, a crucial step in the process of infection and indeed for the survival of the virus [57,58]. With this in mind, we attempted in the present investigation to address the contrasting function of V3, that of the frequent mutations necessary to evade host immune responses, and at the same time to retain the required interaction with coreceptors on the host cell. In this respect, we explored the combined electrostatic potentials of the amino acids in the V3 loop and their distribution in all HIV-1 subtypes, for which the tropism and V3 amino acid sequence are known, in order to exploit canonical rules that might exist.

We have performed electrostatic potential calculations of the gp120 V3 loops, using the Poisson-Boltzmann method [59] and clustering analysis [60] of the spatial distributions of electrostatic potentials for several HIV-1 subtypes. The clustering analysis allows the classification of similarities/dissimilarities of the subtypes based on the common property of electrostatic potentials. Electrostatic interaction is expected because, typically, the V3 loop has an excess of positive charge and the putative interacting N-terminal domain of the coreceptor CCR5, and to a lesser extent CXCR4, has an excess of negative charge. We have performed similar clustering analysis for the spatial distributions of charges and for sequence similarities of HIV-1 subtypes. It is actually the property of charge that many researchers have investigated to shed light into the V3 loop-CCR5/CXCR4 interaction. For example, a recent study has proposed that positively charged amino acids at positions 11, 24 and 25 are involved in coreceptor selection and binding (the "11/24/25" rule [12]). In our study we present an analysis that includes the sequence specificities and charges of V3 loops from various subtypes, but also incorporates the more detailed information that is hidden within the spatial distributions of electrostatic potentials. It is actually the electrostatic potential that is responsible for recognition of two proteins if they have excess of opposite net charges. Recognition, which in our protein-protein interaction model refers to the formation of a weak and nonspecific encounter complex, is followed by binding, which is the formation of the specific final complex [27-30,61-69]. Although the origin of the electrostatic potential is unit and partial charges located in the protein surface and interior, the protein net charge does not capture the effect of charge distribution on protein-protein interactions. It is the spatial distributions of electrostatic potentials of two proteins that mediate long-range electrostatic interactions and protein-protein recognition. It is also the spatial distributions of charges of the two proteins that participate in mediating short-range charge-charge (salt bridging or weak Coulombic effects) and charge-dipole or dipole-dipole (hydrogen bonding) interactions and the formation of the final protein complex. The underlying hypothesis is described by the following transitive argument: if the electrostatic potentials and charges mediate protein-protein association, and if association mediates viral entry, we can deduce correlations to virulence by studying the specific properties of electrostatic potentials and charges, such as type (positive/negative), strength, and spatial distributions. These types of correlations are indications of where to look for causalities and may be helpful in predicting viral attributes.

### Clustering of electrostatic potentials, charges, and sequences

**Figure 2:**
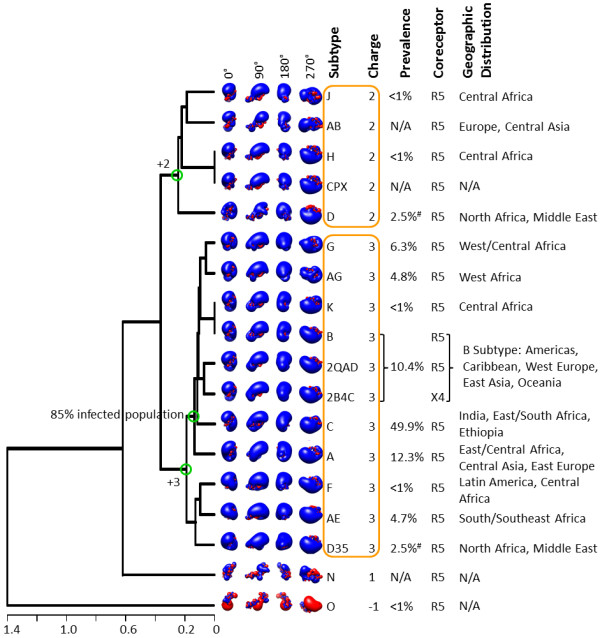
**Electrostatic clustering analysis of the HIV-1 subtypes, using the year 2009 consensus sequences and structural template derived from the gp120 structure with PDB Code **2QAD. The horizontal axis of the dendrogram represents electrostatic similarity distance. Electrostatic potentials were calculated using ionic strength corresponding to 0 mM salt concentration. Isopotential contours are presented in four different orientations, corresponding to rotations about the vertical axis (indicated in the figure). Isopotential contours are plotted at ± 1 k_B_T/e, with blue and red corresponding to positive and negative electrostatic potentials, respectively. The net charge, global prevalence, geographic distribution, and coreceptor selectivity are indicated in the figure for each subtype. N/A denotes that information was not available. The orange boxes highlight clusters with HIV-1 subtypes that have similar electrostatic potential and same charge. Green circles in the branches of the dendrogram denote intersection points between net charges or infected population. The symbol # refers to the global prevalence of Subtype D, which includes D and D35 combined.

Figure 2 shows the dendrogram that clusters the calculated spatial distributions of V3 loop electrostatic potentials. These calculations were performed using 0 mM ionic strength, depicting largest magnitude of Coulombic interactions within each structure which are unscreened by solvent ions. The calculations were performed using homology model structures derived from the crystallographic structure of gp120 with PDB Code 2QAD and the HIV-1 subtype consensus sequences available in the year 2009 at the HIV Databases of the Los Alamos National Laboratory (Table 1). Clustering has been performed by pairwise comparison of the electrostatic potentials of all subtypes listed in Table 1, as described in Methods. V3 loop subtypes with similar spatial distribution of electrostatic potential cluster together. The V3 loops studied have positive net charge, with the exception of group O, which has -1 net charge (Figure 2). The predominant net charge is +3, appearing in 9 subtypes (A, AE, AG, B, C, D35, G, F, K) and in the sequences of the two crystal structures, 2QAD and 2B4C, which belong to subtype B (Figure 2). From the remaining subtypes, group N has a net charge of +1 and AB, D, H, J, and CPX have net charge of +2 (Figure 2). Although subtypes with the same net charge cluster together, there are finer subclusters for subtypes that discriminate according to the spatial distribution of electrostatic potentials. For example, from the +2 subtypes: AB and J cluster together; H and CPX cluster together (they are identical); and D clusters on its own. Overall, the +2 subtypes form the following cluster (with subclusters in brackets/parentheses): {[(J, AB), (H, CPX)], D} (Figure 2). Similarly, the +3 subtypes form the following cluster: {[(((G, AG), (K, B)), (2QAD, 2B4C), C), A], [(F, AE), D35]} (Figure 2). The +2/+3 subtypes form a supercluster together. The +1 group N clusters on its own and forms a larger supercluster with the +2/+3 subtypes, whereas the -1 group O clusters entirely on its own (Figure 2).

**Figure 3:**
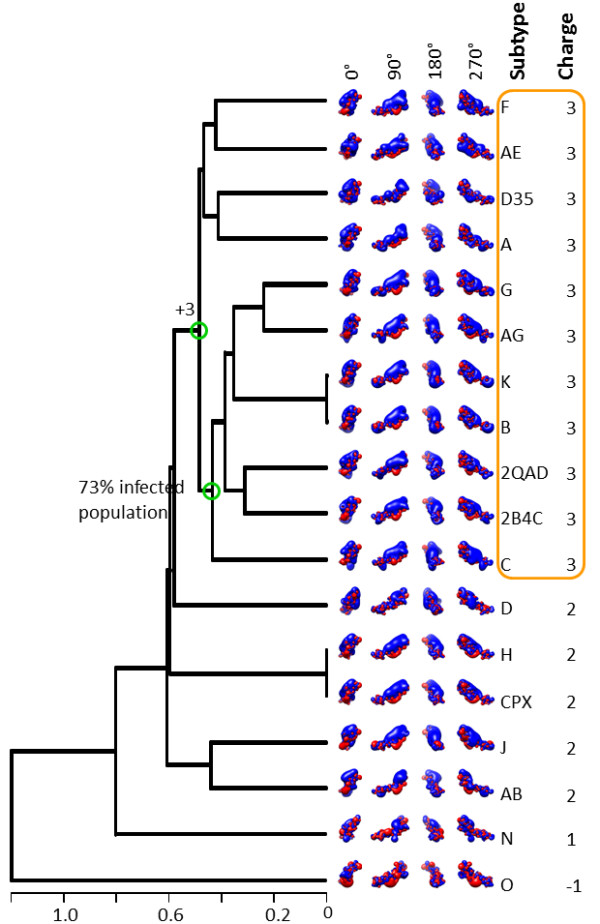
**Electrostatic clustering analysis of the HIV-1 subtypes, using the year 2009 consensus sequences and structural template derived from the gp120 structure with PDB Code **2QAD. The horizontal axis of the dendrogram represents electrostatic similarity distance. Electrostatic potentials were calculated using ionic strength corresponding to 150 mM salt concentration. Isopotential contours are presented in four different orientations, corresponding to rotations about the vertical axis (indicated in the figure). Isopotential contours are plotted at ± 1 k_B_T/e, with blue and red corresponding to positive and negative electrostatic potentials, respectively. The orange box highlights clusters with HIV-1 subtypes that have similar electrostatic potential and same charge. Green circles in the branches of the dendrogram denote intersection points between net charges or infected population.

In a dendrogram, generated with the more realistic electrostatic potential calculations using 150 mM ionic strength (corresponding to physiological ionic strength in serum), we observe similar overall clustering with local variations (Figure 3). For example, the +3 subtypes form the following cluster (with subclusters in brackets/parentheses): {[(F, AE), (D35, A)], [((G, AG), (K, B)), (2QAD, 2B4C)], C}. The +2 subtypes form individual clusters (D), (H, CPX), and (J, AB) within the +2/+3 supercluster. The +1 group N clusters on its own and forms a larger supercluster with the +2/+3 subtypes, whereas the -1 group O clusters entirely on its own (Figure 3). Coulombic interactions within the V3 loops are screened by solvent ions, which results in less obvious differences in the spatial distributions of electrostatic potentials when inspected visually (e.g., compare isopotential contours of Figure 3 to Figure 2). Nevertheless, we observe persistent electrostatic clustering patterns for the various subtypes, despite differences in their V3 loop sequences.

**Figure 4:**
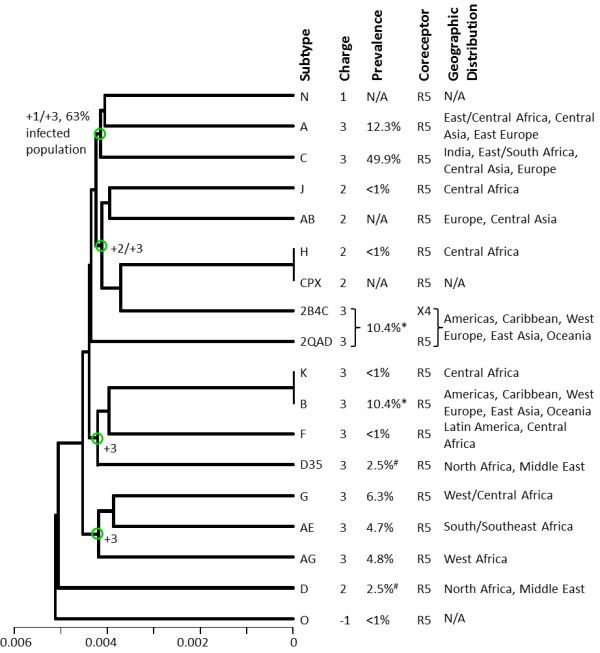
**Charge distribution clustering analysis of the HIV-1 subtypes, from the year 2009 consensus sequences and structural template derived from the gp120 structure with PDB Code **2QAD. The horizontal axis of the dendrogram represents charge similarity distance. The net charge, global prevalence, coreceptor selectivity and geographies of each subtype are indicated in the figure for each subtype. N/A denotes that information was not available. Green circles in the branches of the dendrogram denote intersection points between net charges or infected population. The symbol * refers to the global prevalence of Subtype B which includes the two crystal structural templates (from 2QAD and 2B4C). The symbol # refers to the global prevalence of Subtype D which includes D and D35, combined.

The clustering of the distribution of charges in space for each subtype is shown in Figure 4. Some clusters within this dendrogram can be found in Figures 2 and 3 (e.g., H and CPX). However, the subtypes are mostly mixed within the +1/+2/+3 supercluster. In general, charge distribution does not depict subtle differences between the subtypes. This is because charges are localized in the structure and are independent from each other. However, electrostatic potentials, generated by these charges, have additional features. First, electrostatic potentials account for dielectric and ionic screening. Because of the latter, we observe differences in the magnitudes and shapes of electrostatic potentials in Figures 2 and 3. Second, electrostatic potentials account for spatial enhancements (additive effect of potentials with same signs) or spatial cancellations (subtractive effect of potentials with opposite signs).

**Figure 5:**
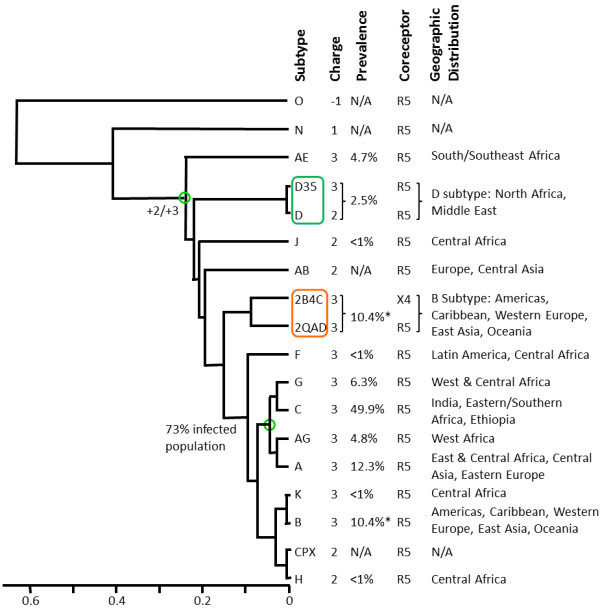
**Sequence clustering analysis of the HIV-1 subtypes, from the 2009 consensus sequences, based on sequence similarity**. The horizontal axis of the dendrogram represents sequence similarity distance. Global prevalence, coreceptor selectivity and geographic distribution of each subtype are indicated in the figure. N/A denotes that information was not available. The green box highlights sequences that belong to Subtype D, while the orange box highlights the two crystal structural templates (from 2QAD and 2B4C), which belong to subtype B. The * refers to the global prevalence of Subtype B which includes the two crystal structure templates.

Figure 5 shows clustering of the sequences of the gp120 V3 loops from the subtypes used to generate the data of Figures 2 and 3. This dendrogram does not, in general, depict the charge or the electrostatic potential differences of the various V3 loops. Obvious examples are the clusters (K, B, CPX, H) and (D35, D) which mix sequences with +2 and +3 net charges. These observations suggest that electrostatic clustering is more detailed, containing more refined charge-related information, than sequence clustering.

### Clustering and epidemiological data

Figures 2 and 3 also present correlations between the observed clusters and available epidemiological data on global prevalence and geographic distribution (year 2004, [21]), and coreceptor selectivity (see below). Subtype C is responsible for almost 50% of the infected population [21]. In the 0 mM data subtype C forms a cluster together with subtypes A, G, AG, K and B, accounting together for ~85% of the infected population (Figure 2). In the 150 mM data subtype C forms a cluster together with subtypes G, AG, K, and B, accounting together for ~73% of the infected population (subtype A, corresponding to ~12.3% of the infected population, moved to a neighboring cluster; Figure 3). Geographic distributions [21] are also quoted in Figures 2 and 4.

### Clustering and structural variability

For many years the intact structure of V3 loop in gp120 was elusive, presumably because of its dynamic character. This was alleviated in the crystal structures 2QAD and 2B4C, which contain multi-protein complexes that stabilize gp120 and the V3 loop. (In both crystal structures, the V3 loop is stabilized by contacting the antibody components of the multi-protein complex.) The dynamic character of the V3 loop can be deduced by observing that its conformation is significantly different in the two crystal structures, 2QAD and 2B4C (Figure 1), despite the fact that they differ only in two conservative mutations (Q/N and F/L, Table 1). To assess the degree that V3 loop dynamics affect its electrostatic properties, at least using two extreme conformations of the crystal structures, we performed similar clustering analyses for electrostatic potentials and charges, using the 2B4C structure (Additional Files 1, 2 and 3). Electrostatic potential clustering at 0 mM ionic strength (Additional File 1) is similar to the corresponding data of the 2QAD structure (Figure 2). However, there are differences in the 150 mM data (Additional File 2 and Figure 3), i.e. +2 subtypes are scrambled within the +3 subtype clusters. The difference between the 150 mM clustering data from the two crystal structures originates from their conformational variability, which results in different charge distributions and different enhancements or cancellations of positive/negative electrostatic potential distributions. Such differences are not observed in the 0 mM data, because of lack of ionic screening, resulting in more uniform distribution of the dominant electrostatic potential (here being positive with the exception of subtype O). As in the case of 2QAD, in 2B4C clustering of spatial distributions of charges does not depict the fine clustering of electrostatic potential similarities/dissimilarities (compare Additional Files 1 and 2). Also, as in the case of 2QAD, in 2B4C electrostatic clustering is more detailed, containing refined charge-related information not present in sequence clustering (compare Additional Files 1, 2 and 3, and Figure 5).

### Influence of homology modeling-derived local flexibility in calculating electrostatic similarity

Our goal in the studies described above was to produce and analyze consensus electrostatic potential templates for the V3 loop structures that capture the average electrostatic characteristics of each consensus sequence. The consensus sequences were constructed using the highest-occurrence amino acid at each V3 loop position, using several thousands of patient sequences. It should be understood that amino acid changes to revert a consensus sequence back to one of the many sequences used to construct the consensus sequence, would affect the V3 loop structure at the vicinity of the change(s), as well as the corresponding electrostatic potential distributions. In addition to sequence variability, the structural flexibility of the V3 loop indicates dynamic electrostatic potential distributions around an average distribution within each subtype.

As mentioned above, with knowledge of the great structural flexibility of the V3 loop, our strategy was to perform our analysis twice using the two crystallographic structures of the V3 loop in order to represent two extremes of the possible conformations and thereby accounting for a conformational transition. Additionally, the analysis based on each crystallographic template was also performed twice, using ionic strengths corresponding to counterion concentrations of 0 and 150 mM, resulting in a total of 4 electrostatic similarity analyses (Figures 2 and 3, and Additional Files 1 and 2). Calculations at 0 mM ionic strength produce electrostatic potentials which are more dispersed and smoother, not as affected by the underlying structure as the 150 mM potentials, whereas calculations at 150 mM potentials, in addition to representing physiological conditions, are more dependent on the underlying structural details.

As a test to assess the effects of local flexibility on the reliability of our electrostatic potential similarity analysis, we produced 5 homology models for each of the two V3 loop sequences corresponding to those of the crystallographic structures. This was made possible with Modeller, by back-predicting structures using the crystallographic template structures from 2B4C and 2QAD. When comparing the 5 homology models to their actual crystallographic template we observe that there is only slight variation, occurring mainly because of different side chain rotamers. We performed electrostatic potential calculations for each set of models at both 0 and 150 mM ionic strength, and computed electrostatic similarities between the electrostatic potentials of each of the 5 homology models and the electrostatic potential of the corresponding template structure. The means and standard deviations of the calculated electrostatic similarities for the models of each template structure at both ionic strengths, are shown in Table 2. It is observed that the electrostatic potentials calculated for the homology models at 0 mM ionic strength were quite similar to those of the template structure, since the mean ESD is ~0.1 for both template structures (Table 2). When looking at the dendrogram of Figure 2, which was calculated at 0 mM ionic strength, we notice that an ESD value of 0.1 is lower than the branches of most clusters, suggesting that such variation is unlikely to significantly affect the overall clustering. When looking at the 150 mM data we observe that the mean ESDs are a little higher at a value of ~0.4, as anticipated given the less smooth and more detailed electrostatic potentials compared to those at 0 mM. However, by analyzing the 150 mM dendrogram in Figure 3, we observe that it is unlikely that these variations would have a dramatic effect on clustering either, since once again the 0.4 value is near the ESD of most pairings. These tests show that the homology modeling procedure does not exactly reproduce the parent potential, but the variations observed are acceptable given the local flexibility of the small V3 loop peptides. A previous study of the effect of homology modeling on electrostatic similarity calculations has concluded that the variation of electrostatic potentials in homology models and deviations from electrostatic potentials corresponding to experimental structures is comparable to electrostatic potential variations within NMR ensembles of structures or within molecular dynamics trajectories [39]. In our case, the consensus electrostatic potentials resulting from homology modeling based on two structural templates and at two ionic strengths provide electrostatic fingerprints that account for sequence variability and structural flexibility. These fingerprints can be used to understand the binding properties of each subtype and to predict the classification of new sequences.

**Table 2:** Comparisons of ESDs of multiple V3 loop homology models.

Structural Template Sequence (Ionic Strength)	Mean ESD	SD
2B4C (0 mM)	0.13	0.04
2QAD (0 mM)	0.10	0.02
2B4C (150 mM)	0.43	0.09
2QAD (150 mM)	0.35	0.06

Each Table entry presents the mean ESD and standard deviation (SD) for 5 homology models generated using Modeller. The modeled structures were constructed by back-prediction using the crystallographic template structures and their sequences (from 2B4C and 2QAD). Electrostatic potentials for each family of 5 homology models were calculated at 0 and 150 mM ionic strength. The goal of this comparison is to assess the effect of structural variability, observed within each family of 5 homology models, on the calculated ESDs.

### Sequence, glycosylation, and charge rules for coreceptor selectivity

**Figure 6:**
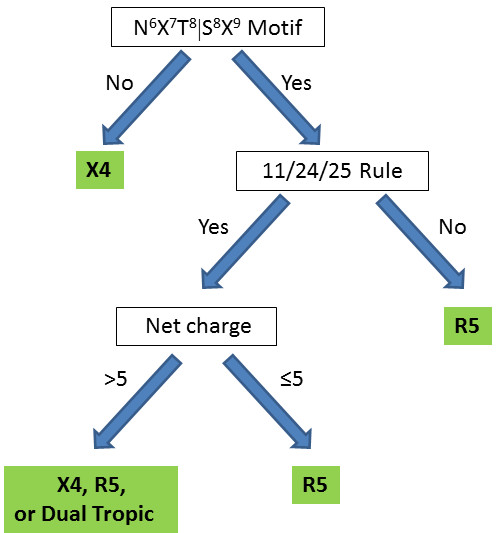
**Flow chart for prediction of HIV-1 coreceptor selectivity based on V3 loop sequence and charge properties**. This scheme is based on the presence of the N^6^X^7^T^8^|S^8^X^9 ^sequence/glycosylation motif [71], the presence of a positive amino acid at sequence positions 11, 24, and 25 (the 11/24/25 rule) [12], and the net charge. The presence of acidic chemical groups in the glycosylation patterns (e.g., sialic acids) could affect the charge of the V3 loop, thus affecting the coreceptor selection. Therefore, the virus can use CXCR4, CCR5 or both receptors for cell entry (dual tropic).

Because there are no X4-tropic consensus sequences in the 2009 data, with the exception of the non-consensus sequence of crystal structure 2B4C (Figure 2), we resorted to sequence, glycosylation, and charge rules to present a predictive scheme for coreceptor selectivity. The coreceptor selection by HIV-1 is known to be influenced by the charge of the V3 loop, amino acid types at specific locations, and the presence of glycosylation sites. Differences in coreceptor selection by HIV-1 subtypes have been shown by experimental studies [12,20,70,71], and computationally predicted [72-76], although the effectiveness of the predictions is not conclusive. Based on previous studies and renewed thinking with respect to net charge, we used several criteria for coreceptor selection, shown in Figure 6. If the glycosylation motif (N^6^X^7^T^8^|S^8^X^9^, where × ≠ Pro and N being the glycosylation site) is absent from the V3 loop sequence, the virus will show preference toward CXCR4 as coreceptor. Experimental studies have demonstrated that loss of glycosylation sites in the V3 loop is associated with selection of CXCR4 [70,71]. If the N^6^X^7^T^8^|S^8^X^9 ^motif is present, the coreceptor selection will be influenced by the amino acids at positions 11, 24, and 25 (of the "11/24/25" rule); if any of these amino acids are not positively charged, the virus will show preference toward CCR5 [12]. We propose that if the N^6^X^7^T^8^|S^8^X^9 ^glycosylation motif is present and any of the amino acids at positions 11, 24 and 25 are positively charged, coreceptor preference will be governed by the net charge of the V3 loop sequence. If the net charge of the V3 loop is > 5, the virus will show preference toward CXCR4. Experimental studies have suggested that a high charge in the V3 is associated with loss of the glycosylation site and utilization of CXCR4 [71]; however if the net charge of the V3 loop is ≤ 5, the virus will show preference for CCR5. Coreceptor selection will be affected by the presence and number of acidic chemical groups, like sialic acids, in the glycans. Typically, the glycans can have up to four sialic acids, each adding one negative charge to the loop [77]. Thus, the presence of glycans may reduce the net charge of sequences with amino acid net charge of > 5 to ≤ 5. This means that a sequence classified as X4-tropic based on amino acid net charge, can be reclassified as R5-tropic using net charge based on amino acids and glycans. Because the number of sialic acids is not known, sequences falling in this category are classified as X4-, R5-or dual-tropic (Figure 6). It should be noted that at lower V3 loop net charges (+3, +4), no effect was seen with alteration of N-glycosylation [71]. In our interpretation, if glycosylation takes place, it lowers the net positive net charge even more and thus the sequence remains within the R5-tropic definition according to the scheme of Figure 6. We have tested the flow chart of Figure 6 with experimental data for a series of R5- and X4-tropic sequences [70,71] and found consistency between the predicted and experimentally-derived tropisms. All consensus sequences studied here, and the sequence of 2QAD crystal structure, are R5-tropic according to the scheme of Figure 6, perhaps because CCR5 is the first viral preference for the asymptomatic cell infection prior to switching to CXCR4, and an insufficient number of X4-tropic sequences is available for consensus. However, individual patients infected with X4-tropic viruses of the aforementioned data of Refs. [70,71] have V3 loop sequences which are classified as X4-tropic using the scheme of Figure 6. It is likely that as CCR5 receptors are being depleted, the virus evolves through mutational pressure in increasing the positive charge of the V3 loop for more efficient recognition of cells with CXCR4 receptors. This may be because the N-terminal domain of CXCR4 has smaller negative net charge (and electrostatic potential) than that of CCR5, thus requiring larger positive net charge (and electrostatic potential) in the V3 loop for interaction.

## Conclusions

In overview, we have performed clustering analysis to distinguish the electrostatic contributions to recognition and binding for the 2009 consensus sequences of the V3 loop of HIV-1 gp120. Our analysis is based on a two-step association model, which distinguishes recognition (formation of a weak nonspecific encounter complex) from binding (formation of a strong specific final complex). Clustering of spatial distributions of electrostatic potentials (in the protein exterior and interior) depicts the significance of long-range electrostatic interactions to the recognition of the V3 loop with extracellular loops of CCR5/CXCR4. Clustering of spatial distributions of charges (in the protein surface and interior) provides information on the significance of individual charges in short-range electrostatic interactions to the binding of the V3 loop to CCR5/CXCR4. This analysis clusters the V3 loop consensus sequences according to the similarities/dissimilarities of their electrostatic potentials and charges. Although clustering of charges and electrostatic potentials share similarities, they are in general different with the former emphasizing local effects and the latter emphasizing macroscopic effects. In addition, electrostatic potentials are sensitive to ionic strength effects, which is not the case for charges. This type of clustering, at the level of the specific physicochemical property, is not depicted in the widely used clustering of sequences, although conceptually sequences are closer to charges as they contain alignments of amino acids with specific physicochemical properties, including charge. The major advantage of charges and electrostatic potentials is that they contain information of spatial physicochemical details, which is not present in sequences.

Clustering of charges and electrostatic potentials provides a refined analysis, compared to clustering of sequences, for proteins in which electrostatics is the driving force for association, as is the case of the gp120 V3 loop. The clustering of electrostatic potentials is of particular importance for inhibitor design and eventually for anti-HIV drug design. As we have shown previously for the case of short peptides derived from the V3 loop of gp120, scrambling of charges within the sequence does not affect binding to an N-terminal peptide of CCR5 or inhibition in infectivity assays [18,19]. The magnitude of the electrostatic potential was in general proportional to net charge for highly positively charged V3 loop-derived peptides (with additive electrostatic potential property), and correlated well with binding and inhibition data. In the case of the flexible and variable V3 loop, targeting the recognition process, and specifically targeting the bulk physiochemical property of the electrostatic potential, may be an efficient avenue for drug design. This may be possible as long as the spatial distribution of the electrostatic potential remains largely invariable despite the dynamic character of the V3 loop. In the present study, we provide a database of electrostatic property classification for consensus sequences of gp120, at the V3 loop level, for the time point of year 2009. We also provide correlations with prevalence and geographic distribution and coreceptor selectivity. Coreceptor selectivity depends on the specific N^6^X^7^T^8^|S^8^X^9 ^sequence motif, the specific positive charge location according to the 11/24/25 rule, and the overall charge and electrostatic potential distribution mediated not only by charged amino acid side chains, but also by glycosylation patterns. For this reason, an elaborate scheme for determining coreceptor selectivity is presented.

## Abbreviations

HIV: human immunodeficiency virus; gp120: glycoprotein 120; gp41: glycoprotein 41; CD4: cluster of differentiation 4; CCR5: chemokine receptor 5; CXCR4: chemockine receptor 4 with CXC motif; R5: selection of CCR5 as coreceptor; X4: selection of CXCR4 as coreceptor; CCR5-Nt: N terminal extracellular domain of CCR5; ECL2: extracellular loop 2; CRF: circulation recombinant forms; PDB: Protein Data Bank; ESD: electrostatic similarities distance.

## Authors' contributions

ALdV carried out the calculations and data analysis and participated in drafting the manuscript. CAK assisted with the calculations and electrostatic analysis. AKR, EK and DM conceived the idea for the role of electrostatics in V3 loop binding and participated in drafting the manuscript. DM supervised the design of the study. All authors contributed in the scientific discussions that resulted in the proposed model. All authors read and approved the final manuscript.

## Supplementary Material

Additional File 1 **Electrostatic potential clustering analysis of the HIV-1 subtypes, from the 2009 consensus, using the structure with PDB Code **2B4C**as template**. The horizontal axis of the dendrogram represents electrostatic similarity distance. Electrostatic potentials were calculated using ionic strength corresponding to 0 mM salt concentration. Isopotential contours are presented in 4 different orientations, corresponding to rotations about the vertical axis. Isopotential contours are plotted at ± 1 k_B_T/e, with blue and red corresponding to positive and negative electrostatic potentials, respectively. The net charge, global prevalence, coreceptor selectivity, and geographic distribution are indicated in the figure for each subtype. N/A denotes that information was not available. The orange boxes highlight clusters with HIV-1 subtypes that have similar electrostatic potential. Green circles in the branches of the dendrogram denote intersection points between net charges or infected population. The * refers to the global prevalence of subtype B, which include the two structural templates (2B4C and 2QAD). The # refers to the global prevalence of subtype D, which include D and D35. Click here for file

Additional File 2 **Electrostatic potential clustering analysis of the HIV-1 subtypes, from the 2009 consensus, using the structure with PDB Code **2B4C**as template**. The horizontal axis of the dendrogram represents electrostatic similarity distance. Electrostatic potentials were calculated using ionic strength corresponding to 150 mM salt concentration. Isopotential contours are presented in 4 different orientations, corresponding to rotations about the vertical axis. Isopotential contours are plotted at ± 1 k_B_T/e, with blue and red corresponding to positive and negative electrostatic potentials, respectively. The orange box highlight clusters with HIV-1 subtypes that have similar electrostatic potential and same charge. Green circles in the branches of the dendrogram denote intersection points between net charges or infected population. Click here for file

Additional File 3 **Charge distribution clustering analysis of the HIV-1 subtypes, from the 2009 consensus, using the structure with PDB Code **2B4C**as template**. The horizontal axis of the dendrogram represents charge similarity distance. The net charge, global prevalence, coreceptor selectivity and geographical distribution are indicated in the figure for each subtype. N/A denotes that information was not available. Green circles in the branches of the dendrogram denote intersection points between net charges or infected population. The * refers to the global prevalence of subtype B, which include the two structural templates (2B4C and 2QAD). The # refers to the global prevalence of subtype D, which include D and D35. Click here for file
