# Supplementary figures and images for: Clustering of HIV-1 Subtypes Based on gp120 V3 Loop electrostatic properties
Source: BMC Biophys. 2012 Feb 7;5:3. doi: 10.1186/2046-1682-5-3 (PMC3295656; doi:10.1186/2046-1682-5-3)

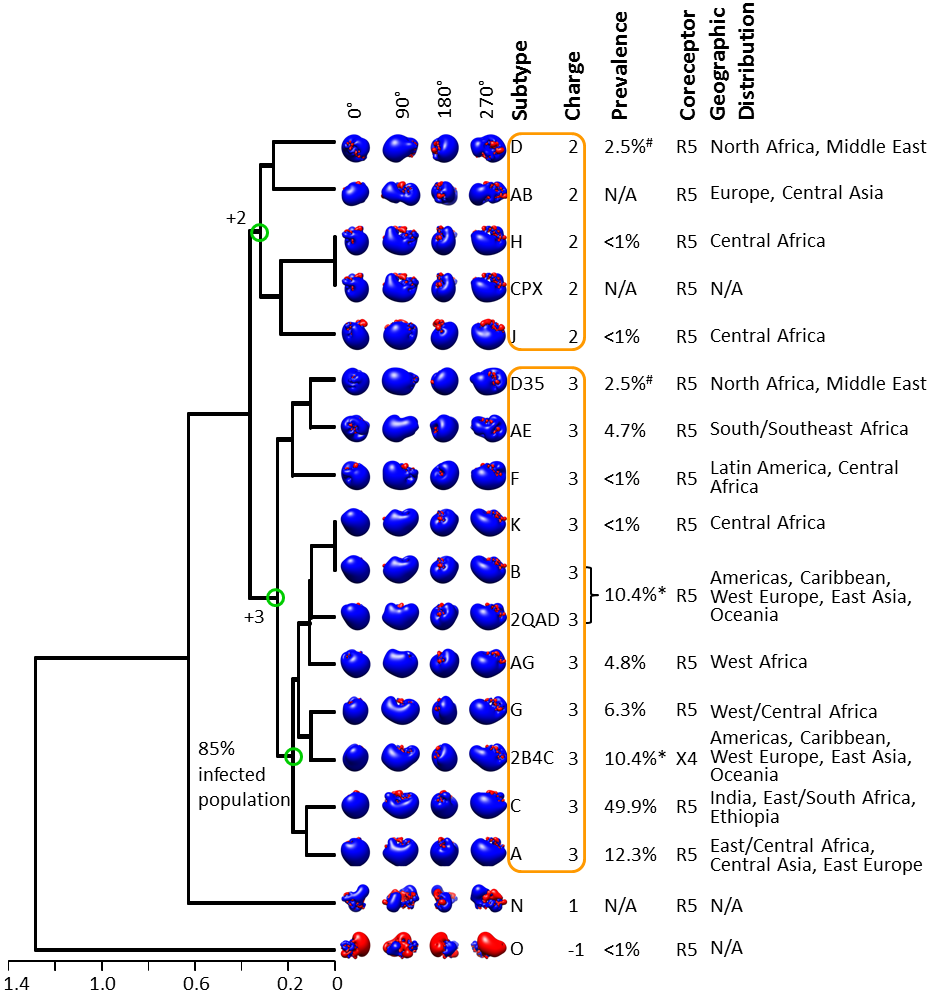

Supplement: Additional File 1 — Electrostatic potential clustering analysis of the HIV-1 subtypes, from the 2009 consensus, using the structure with PDB Code 2B4Cas template. The horizontal axis of the dendrogram represents electrostatic similarity distance. Electrostatic potentials were calculated using ionic strength corresponding to 0 mM salt concentration. Isopotential contours are presented in 4 different orientations, corresponding to rotations about the vertical axis. Isopotential contours are plotted at ± 1 kBT/e, with blue and red corresponding to positive and negative electrostatic potentials, respectively. The net charge, global prevalence, coreceptor selectivity, and geographic distribution are indicated in the figure for each subtype. N/A denotes that information was not available. The orange boxes highlight clusters with HIV-1 subtypes that have similar electrostatic potential. Green circles in the branches of the dendrogram denote intersection points between net charges or infected population. The * refers to the global prevalence of subtype B, which include the two structural templates (2B4C and 2QAD). The # refers to the global prevalence of subtype D, which include D and D35. [file 2046-1682-5-3-S1.PNG]

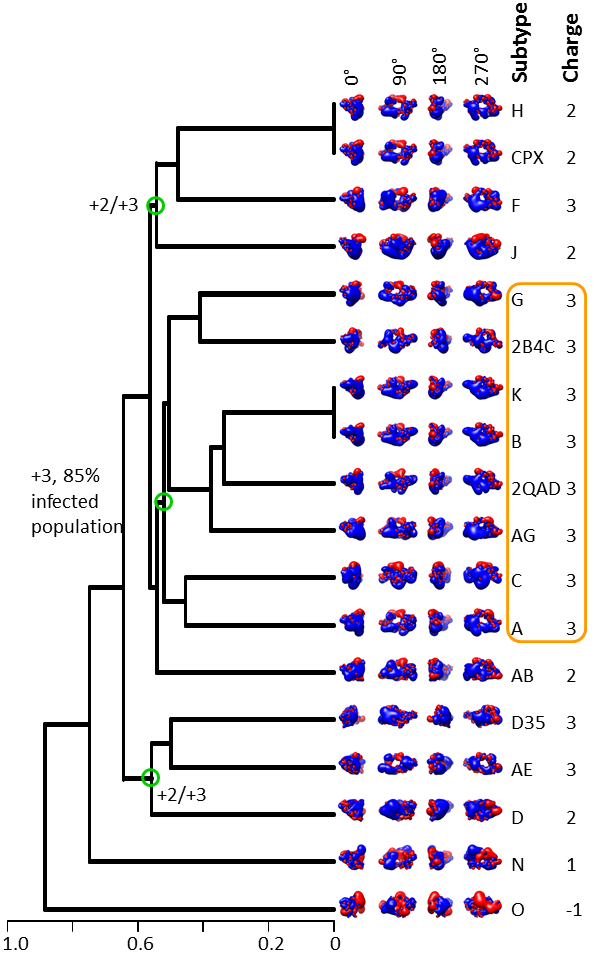

Supplement: Additional File 2 — Electrostatic potential clustering analysis of the HIV-1 subtypes, from the 2009 consensus, using the structure with PDB Code 2B4Cas template. The horizontal axis of the dendrogram represents electrostatic similarity distance. Electrostatic potentials were calculated using ionic strength corresponding to 150 mM salt concentration. Isopotential contours are presented in 4 different orientations, corresponding to rotations about the vertical axis. Isopotential contours are plotted at ± 1 kBT/e, with blue and red corresponding to positive and negative electrostatic potentials, respectively. The orange box highlight clusters with HIV-1 subtypes that have similar electrostatic potential and same charge. Green circles in the branches of the dendrogram denote intersection points between net charges or infected population. [file 2046-1682-5-3-S2.PNG]

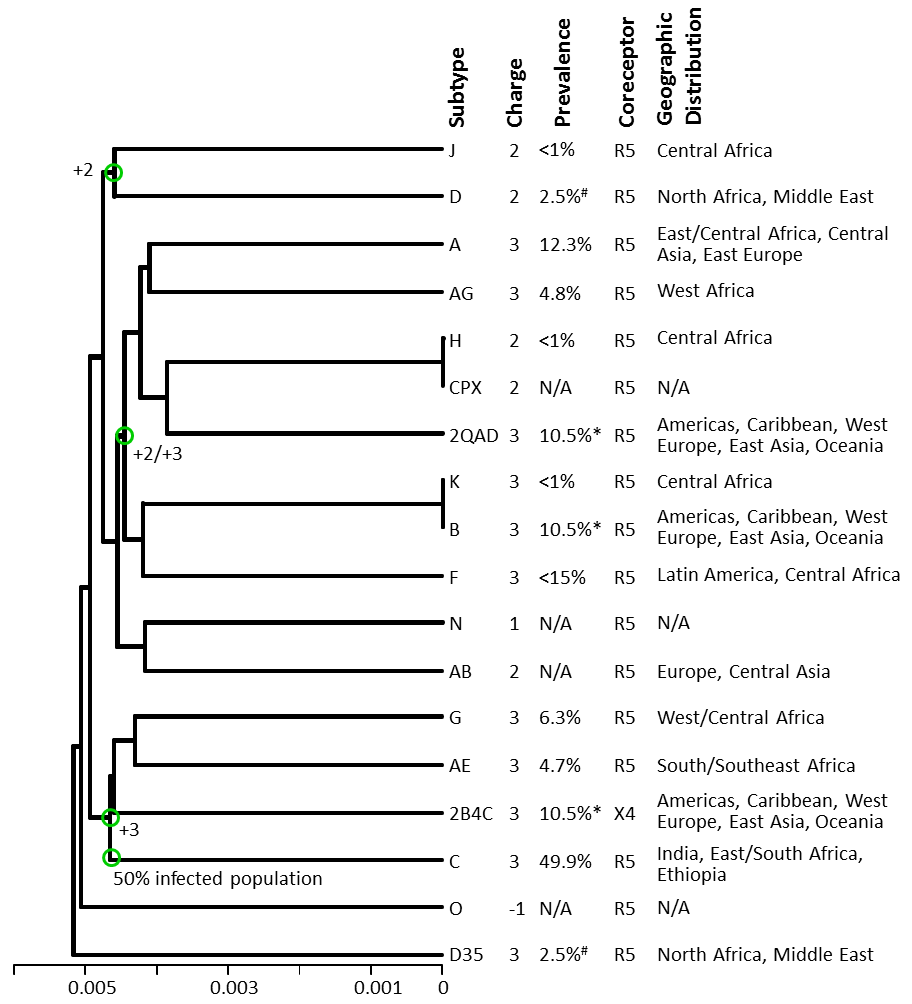

Supplement: Additional File 3 — Charge distribution clustering analysis of the HIV-1 subtypes, from the 2009 consensus, using the structure with PDB Code 2B4Cas template. The horizontal axis of the dendrogram represents charge similarity distance. The net charge, global prevalence, coreceptor selectivity and geographical distribution are indicated in the figure for each subtype. N/A denotes that information was not available. Green circles in the branches of the dendrogram denote intersection points between net charges or infected population. The * refers to the global prevalence of subtype B, which include the two structural templates (2B4C and 2QAD). The # refers to the global prevalence of subtype D, which include D and D35. [file 2046-1682-5-3-S3.PNG]
